# The influence of heteroatom doping on local properties of phosphorene monolayer

**DOI:** 10.1038/s41598-021-98014-8

**Published:** 2021-09-16

**Authors:** Artur P. Durajski, Konrad M. Gruszka, Paweł Niegodajew

**Affiliations:** 1 Institute of Physics, Czestochowa University of Technology, Ave. Armii Krajowej 19, 42-200 Czestochowa, Poland; 2 Institute of Thermal Machinery, Czestochowa University of Technology, Ave. Armii Krajowej 21, 42-200 Czestochowa, Poland

**Keywords:** Condensed-matter physics, Electronic properties and materials

## Abstract

New energy storage technologies that can serve as a reliable alternative to lithium-ion batteries are in the spotlight. Particular attention has been recently devoted to magnesium-ion systems due to the considerable abundance of this element and also due to its promising electro-chemical performance. Our results show that monolayer black phosphorene doped by B, Sc, Co, and Cu atoms possesses good structural stability with the minimal cohesive energy of $-5.563$ eV/atom, the adsorption energy per Mg atom ranging from $-1.229$ to $-1.357$ eV, and the charge transfer from double-side adsorbed single Mg-ions to the B-substituted phosphorene increased by $\sim$0.21 $e^-$ in comparison with pristine phosphorene. The present work demonstrates a potential path for future improvements of phosphorus-based anode materials for Mg-ion rechargeable batteries which were evaluated using first-principles density-functional theory calculations.

## Introduction

Enormous development, production, and deployment of a variety of portable electronic devices trigger a number of problems that need to be faced by the world energy sector. One of them is a continuous need to improve the energy capacity, efficiency, and life voltage of rechargeable solid-state batteries (SSB). In applications such as portable devices or electric vehicles, lithium-ion batteries have currently no contender in terms of energy density or durability^1^. It is important to note that commercially used energy storage systems utilizing mainly graphite-based anodes and lithium as an electrolyte offer a storage capacity up to 372 mAh/g^1–5^. Among others, silicon would seem to be a possible alternative for the graphite anode because its theoretical capacity is higher. However, the large capacity fade observed during initial cycling has prevented the silicon anode from being commercialized in Li-ion batteries^6^. In turn, the global demand for lithium together with its price is expected to rise dramatically in the coming years^7–10^. These trends are accompanied by a considerable effort aimed at identifying high-performance materials that can constitute a reliable alternative to lithium^11^. Promising seems to be the use of such monovalent cations as K$^{+}$, Na$^{+}$ or multivalent as Ca$^{2+}$, Mg$^{2+}$ in part due to their rich resources in contrast to lithium^12–14^. However, the development of new non-lithium systems poses a number of unique challenges that need to be overcome such as the large ionic size of the respective ions especially for Na and K systems, weak kinetics, the low-voltage window of Mg-ion systems, etc. Hence, developing efficient electrode materials with novel morphologies being able to cooperate with inexpensive electrolytes is a challenging task, however, it is the only way to harness the potential on the non-Li-ion systems. Today’s progress in nanotechnology, especially the successful fabrication of two-dimensional (2D) materials motivated scientists to investigate (mainly numerically) their electronic performance^15–21^ even though there is no reliable and scalable production technique for high-quality monolayer films^22^. 2D materials possess a unique set of properties not otherwise found in their bulk counterparts - for instance, the bandgap of black phosphorus (BP) increases with a decreasing number of layers and reaches its maximum for the single-layer^23^.

The available literature has already identified the most promising candidates that may serve as electrodes in non-lithium systems, which among others are phosphorene or graphene^24–26^. Particularly interesting is the former one, especially because as it was indicated in^27^ the elemental phosphorus can form Na$_3$P with an outstanding theoretical capacity of 2600 mAh/g while the amorphous red phosphorus/carbon composite offers a reversible storage capacity of 1890 mAh/g^28^. Some first-principle studies^29^ showed that absorption of Na$^{+}$ ions on both sides of the phosphorene layer is more favorable than on a single side. More detailed research on the adsorption process of 20 different adatoms (mainly metals) on phosphorus monolayer showed that it forms strong bonds with all investigated adatoms and still preserves its structural integrity in contrast to such monolayer materials as graphene and silicene^30^. Na-ions diffuse much faster in the layered structures than Li-ions due to much narrower diffusion barrier of sodium 40–63 meV compared to 0.76 eV for lithium^31^, however, Li atoms adsorbed on phosphorene exhibits a higher specific capacity than Na atoms^32^. Some of the recent works were also devoted to studying the volumetric expansion and specific diffusion of Mg ions on the phosphorene surface. Han and co-workers^33^ found that the diffusion of Mg on phosphorene is highly anisotropic with diffusion along the zigzag direction being highly energetically favorable. The more broad approach to the topic of research progress in layered phosphorus can be also found in the recent review paper by Liu et al.^34^. Some other works focus on studying the impact of substitutional and adsorption doping and impurities effects on the performance of BP^35^. The indirect doping effect in BP by different types of vacancies in the h-BN supporting layer studied in^36^ showed that defects in the substrate can act as doping to the BP layer. What is more, p-type doping occurred in most of the analyzed configurations with one exception where n-type doping was observed under isolated nitrogen-vacancy. Substitutional 3d transition metal impurities introduced into black and blue phosphorene layered materials revealed that the largest bandgap (among the doped materials) is obtained when the Sc was used as doping element. In the work^37^ authors showed that oxy-functionalized phosphorene can be automatically formed under either low or high concentration of O$_2$ while the formation of the imine-functionalized phosphorene sheet requires a high concentration of N$_2$H$_2$. In another similar work^38^ it was found that enhancement of adsorption and diffusion of sodium can be achieved by introducing certain defects on BP sheet such as mono-vacancy, di-vacancy, and Stone-Wales. The bandgaps of P-O-half, P-O, P-NH-half, and P-NH functionalized phosphorene monolayers were observed to be equal to 1.55 eV, 1.03 eV, 1.44 eV, and 1.24 eV, respectively. A first principle study performed in^39^ showed that complete fluorination of phosphorene ensures a bandgap of about 2.27 eV. Interesting is also that the bandgap of four-layer BP can be effectively modulated from 0.0 to 0.6 eV when exposed to an electric field generated from the ionized K atoms. There was also an attempt to couple phosphorene with graphene^40^ and it was found that sodiation takes place within a two-step process of intercalation and alloying with the resulting specific capacity of 2440 mAh/g. In another similar work^41^ authors enhanced functionalized BP by bridging it covalently on graphene what improved the stability during the long-cycle operation of the sodium-ion battery and ensured the specific capacity of 1472 mAh/g. Not all papers concerning doped phosphorene are oriented on batteries, as this material can be widely used in other applications such as catalyst for CO oxidation^42^ when phosphorene is doped using Cu, Sc-doped hydrogen sensors^43^, a water treatment solutions by Fe doped phosphorene as shown by Cortes-Arrigada et al.^44^, Al or Cu doped phosphorene uptake of formaldehyde^35^ and optoelectronic NLO properties by superalkali doping in the work by Hanif et al.^45^.

As the above survey shows, the attracted attention of BP as a candidate for anode material in SSBs seems justified. Still, however, other promising solutions are waiting to be discovered either experimentally or numerically. Up to now, no much attention has been devoted to BP operating with Mg-ion systems, which seems to be a good candidate mainly due to its abundance but requires some effort to improve electrode properties. There are several aspects to improve the performance parameters of batteries. Some of them concern about the reduction of the diffusion barrier of adsorbed ions, which may have a positive effect both on the capacity and the working current that can be provided by the cell, others focus on extending the life cycle of the cells mainly through self-regeneration processes. Most of the works deal with the search for structures that can accumulate many ions on their surface, thus improving their capacity. For black phosphorus batteries, which are the subject of the current study two major drawbacks can be established, namely short cell life due to its degradation and relatively low capacitance^31^. In this paper, we aim to improve the latter one, by introducing substitutions to pristine phosphorene. However, in our assumptions those substitutions must meet certain conditions, in particular, they should not lead to a structure destabilization; they should be abundant; they should improve the capacity of the battery. The last of these requirements may be realized in basically two ways: (I) by modification of local structure in such a way, that overall electrode area increases, (II) by reduction of ionic repulsion between adsorbed Mg ions or (III) by influencing of charge transfer to the electrode. Based on those assumptions we choose several atomic substitutions, namely with B, F, Cl, Sc, Co, and Cu ions which we examine in this context. The choice of these substitutions as candidates was dictated by additional criteria: the substitution should not cause induction of local magnetic moments that could impede diffusion; the selected ions should be as light as possible (among those not studied) so as not to deteriorate the capacity per gram ratio heavily; the electron valence configuration of dopant should be possibly different from P atom, to ensure as much impact on the material as possible and therefore represent broad spectrum of additives. As further research revealed, not all of them can meet these requirements.

## Computational methods

Following the methodology utilized, inter alia, in papers^29–31,33,46^, our calculations will be carried out using a supercell approach in which we place a single layer of black phosphorus. This type of single-layer approach obviously has the disadvantage of ignoring effects affecting the operation of the battery under experimental conditions, especially the well-known swelling (volumetric expansion^47^) during multiple charge/discharge cycles, but this work is not intended to investigate these phenomena. However, in our case, we focus mainly on the local effects of introducing atomic additives and study the strict interactions of these additives with the rest of the material plane as well as with adsorbed Mg ions. This approach significantly reduces the computational cost. At the same time, we can focus on more localized electronic interactions which enable an in-depth analysis of those local effects that would not have a direct impact on the mentioned battery degradation anyway.

First-principles calculations were conducted in the framework of the density-functional theory (DFT)^48^ which is embedded in the Quantum Espresso package^49,50^. The generalized gradient approximation of Perdew-Burke-Ernzerhof (GGA-PBE) was used for the exchange-correlation functional together with the projector-augmented wave (PAW) method. The kinetic energy cutoff for the wavefunction and the kinetic energy cutoff for charge density were fixed at 70 Ry and 700 Ry, respectively. The model of pristine is constructed of a $3\times 4$ supercell of phosphorene (containing 48 P atoms). To avoid potential interaction between adjacent phosphorene layers, a vacuum layer of 30 Å was considered. Application of the Broyden-Fletcher-Goldfarb-Shanno (BFGS) quasi-Newton algorithm^51^ ensured the lattice constants and the atomic positions to be fully relaxed during the geometry optimization until the residual forces acting on the atoms remain smaller than 0.001 eV/Å and the total energy change is smaller than $10^{-5}$ eV. The Brillouin zone was sampled with the use of a $5\times 5\times 1$ **k**-mesh in the Monkhorst-Pack scheme. A $30\times 30\times 1$ Monkhorst-Pack grid was applied for the calculations of the electronic band structures and density of states (DOS). The non-doped structure and the B, F, P, Cl, Sc, Co, and Cu doped structures were each time fully relaxed (including optimization of cell size in non-vacuum directions), i.e. after the incorporation of the additive and after the absorption of Mg.

## Results and discussion

The investigation begins with optimization of the geometrical structure of the initial structure of the pristine BP monolayer. Just as in graphene, phosphorus atoms are arranged in a hexagonal lattice, however, unlike graphene the phosphorene layer is not flat due to the sp$^3$ hybridization. Our results showed that the optimized parameters for a free-standing pristine puckered phosphorene monolayer are: $a=4.63$ Å, $b=3.30$ Å and thickness of a single layer is 2.11 Å, which are consistent with the previous reports^30,31,52–54^.

**Figure 1:**
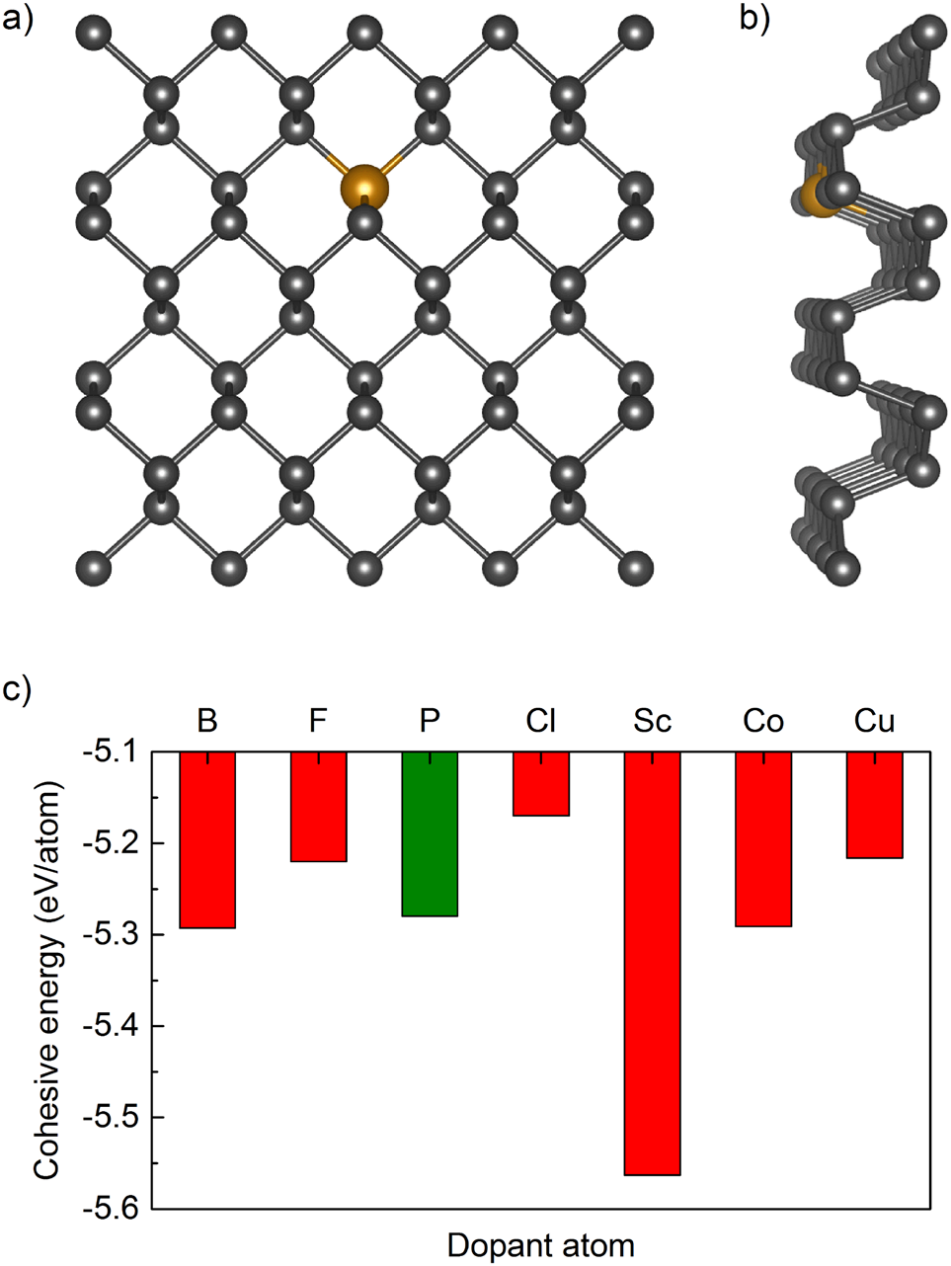
Schematic illustration of the geometrical structure of black phosphorus $3\times 4$ supercell substitutionally doped: (**a**) top view and (**b**) perspective view. Gray and orange balls represent phosphorus and dopant atoms, respectively. (**c**) The cohesive energy of phosphorene (P) doped by B, F, Cl, Sc, Co, and Cu atoms.

**Figure 2:**
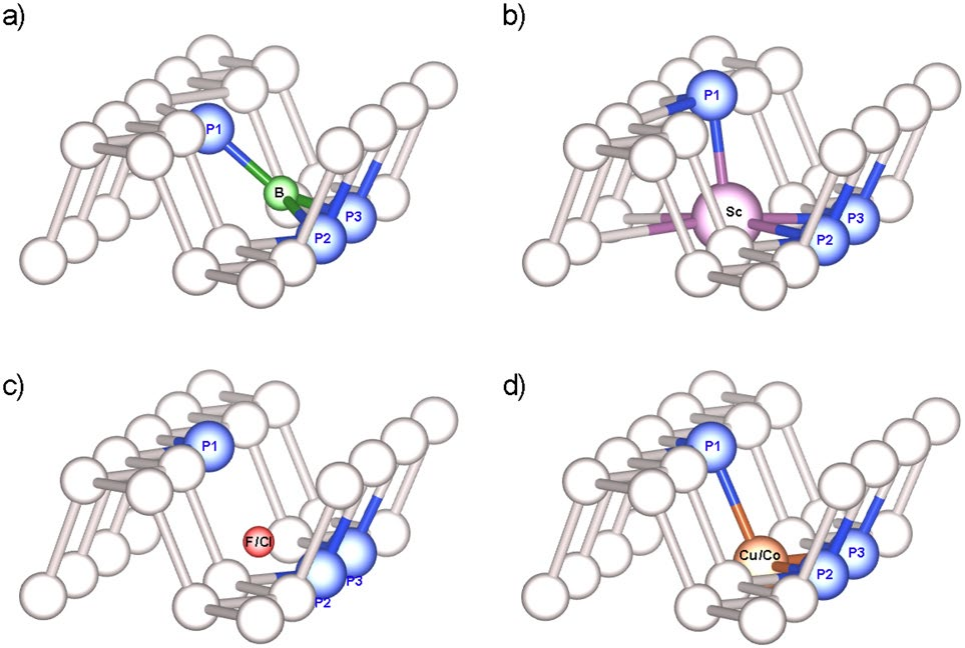
Structural deformation of the (**a**) B, (**b**) Sc, (**c**) F/Cl (**d**) Cu/Co-doped phosphorene lattice.

The influence of a single dopant on the geometrical structure and total energy was investigated using a large $3\times 4$ supercell containing 48 P atoms. Figure 1a and b show the ball-and-stick model of atomic structure for doped phosphorene. Since pure phosphorene consists of non-magnetic elements, it has no magnetic moment. However, many previous reports revealed that a magnetic state could be induced in nonmagnetic 2D systems by defect, substitution, or doping^55–57^. The application of modified systems in energy storage devices is limited due to the magnetic moment. Therefore, the selection of such dopants as B, F, Cl, Sc, Co, and Cu was manifested by the fact that they do not induce magnetism in phosphorene^58^. It is important to note that single doping does not affect the whole crystal lattice, nonetheless, it induces local bond length deformations which scale depends on dopant atom^59–61^.

The binding between P in the pristine phosphorene is due to the $sp^{3}$ hybridization. During the substitution, the breaking of chemical symmetry induces local changes in adjacent atoms positions as the equilibrium is disturbed. After the structure optimization, four types of substitution influence on its near environment were identified, namely: contraction, swelling, drift, and no change as presented in Fig. 2. In three out of four cases a dopant atom tends to be closer to the P2 and P3 phosphorus atoms as they tend to pull the substitution more closely to P1. In the case of B substituted material (Fig. 2a) the contraction effect occurs. The P atom lying in the upper plane (P1) is slightly pulled toward the substitution atom, however, one may observe a much larger displacement of B atom. Such an effect is not surprising as B is both smaller and lighter than P. As can be seen, B is lifted up to the position ensuring an almost in-plane arrangement with P1, P2, and P3. Dihedral angles P1-B-P3 and P1-B-P2 are equal to $\sim$123.6$^{\circ }$ while the remaining P2-B-P3 is $\sim$109$^{\circ }$. This suggests a distorted $sp^{2}$ type hybridization. Consequently, the nearest material structure becomes distorted, but the range of this modification is limited mainly to the relocation of P1 atom. In the Sc case—a swelling representative—(Fig. 2b), the greatest visible influence again applies to P1. This time, however, the substituted atom stays in-plane with the bottom P-zigzag chain and it is slightly pushed into a hollow site further from P2 and P3 atoms. A pushing-out affect of dopant on neighboring P atoms lying in a bottom zigzag plane can be observed as well. As the Sc is the largest in size among introduced substitutions, its swelling-like behavior is somehow expected. Bonding, in this case, resembles distorted square pyramidal with $sp^{3}d^{2}$ type hybridization of the central atom. For F and Cl (Fig. 2c), a drifting behavior of substituted atoms is visible. In structure relaxation, F and Cl atoms drifted below the bottom zigzag chain and tended to bond only with one P atom. Both, Cl and F are very reactive. In most cases their electron affinity in complexes is very strong, pulling a single electron, to close their shell. For this reason both usually bind very strongly to one neighbour when their environment is not fully symmetric. It should be emphasized that only these two substituted atoms are pulled out from BP plane when Mg is adsorbing onto its surface. In the last two cases with Cu and Co substitutions (Fig. 2d) only minor distortions relative to pristine BP were observed.

**Figure 3:**
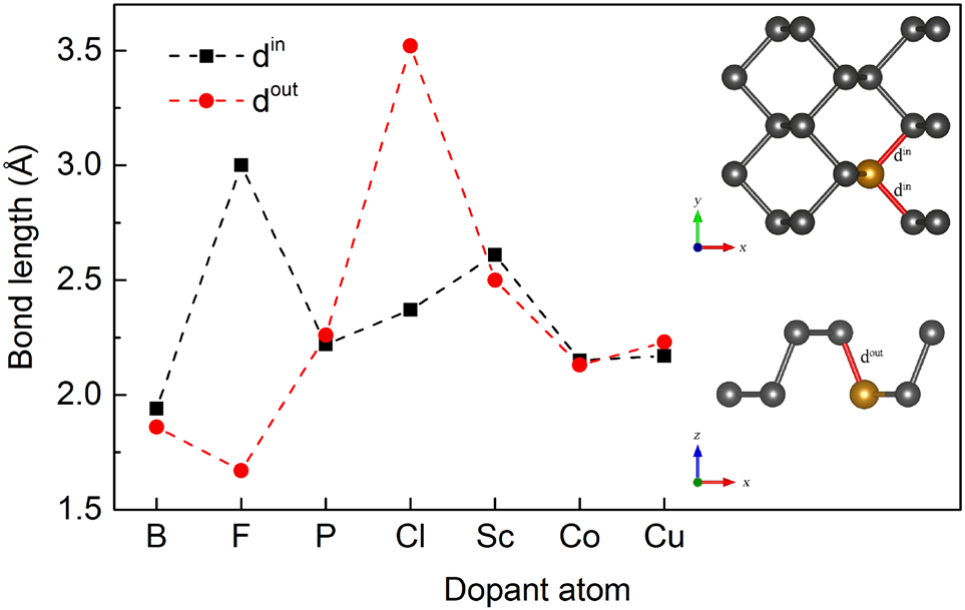
Bond length between P and dopant atom in the xy plane ($d^{in}$) and out of the xy plane ($d^{out}$) for phosphorene (P) substitutionally doped by B, F, Cl, Sc, Co and Cu atoms.

The bonding lengths of doping atoms with nearest neighbor P atoms in the xy plane ($d^{in}$) and out of the xy plane ($d^{out}$) in the phosphorene lattice were examined and the obtained results are plotted in Fig. 3.

For comparison, the calculated P-P bond lengths $d^{in}$ and $d^{out}$ of pristine single-layer black phosphorene are 2.22 and 2.26 Å, respectively. One can see that the smallest deviations from the ground state phosphorene structure are also obtained for systems with the Cu- and Co-substitution. In Fig. 3 it can be also seen that one of the bonds $d^{in}$ in case of F and $d^{out}$ for Cl is significantly elongated, while for the rest of dopants $d^{in}$ and $d^{out}$ bond lengths are nearly equal. This behaviour is due to a significant shift of the F and Cl atoms in relation to their initial position (described previously as the drifting behavior). Because remaining dopants were relatively stable in their position (at the position of the substituted phosphorus) their bond lengths are nearly symmetric.

Having optimized geometries one may next calculate the cohesive energy per atom:

1 $$\begin{aligned} E_{coh}=(E_{\mathrm{P_{47}}+dopant}-n_{i}E_{i})/n, \end{aligned}$$

where $E_{\mathrm{P_{47}}+dopant}$ and $E_{i}$ represent the total energies of the considered substitutionally doped system and the energy of an individual element in the same supercell, respectively. The symbol *n* represents the total number of atoms in the system and *i*={P, B, F, Cl, Sc, Co, Cu}. A value of $E_{coh}$ indicates the energetic stability of the systems. The smaller the value, the more stable the system. The calculated cohesive energies of Cl-, Cu-, F-, Co-, B-, and Sc-doped phosphorene are $-5.170$, $-5.216$, $-5.220$, $-5.291$, $-5.293$, $-5.563$ eV/atom, respectively. Negative values obtained for all substitutions indicate that all configurations are possible. Figure 1c shows the cohesive energy calculated for doped phosphorene. The lowest calculated cohesive energy is for Sc doping when compared to the pristine phosphorene sheet ($E_{coh}=-5.280$ eV/atom). Therefore, if the environment is rich in all of the studied substitution atoms, the Sc would be the most prone to incorporate, due to the criterion for minimum-energy.

**Figure 4:**
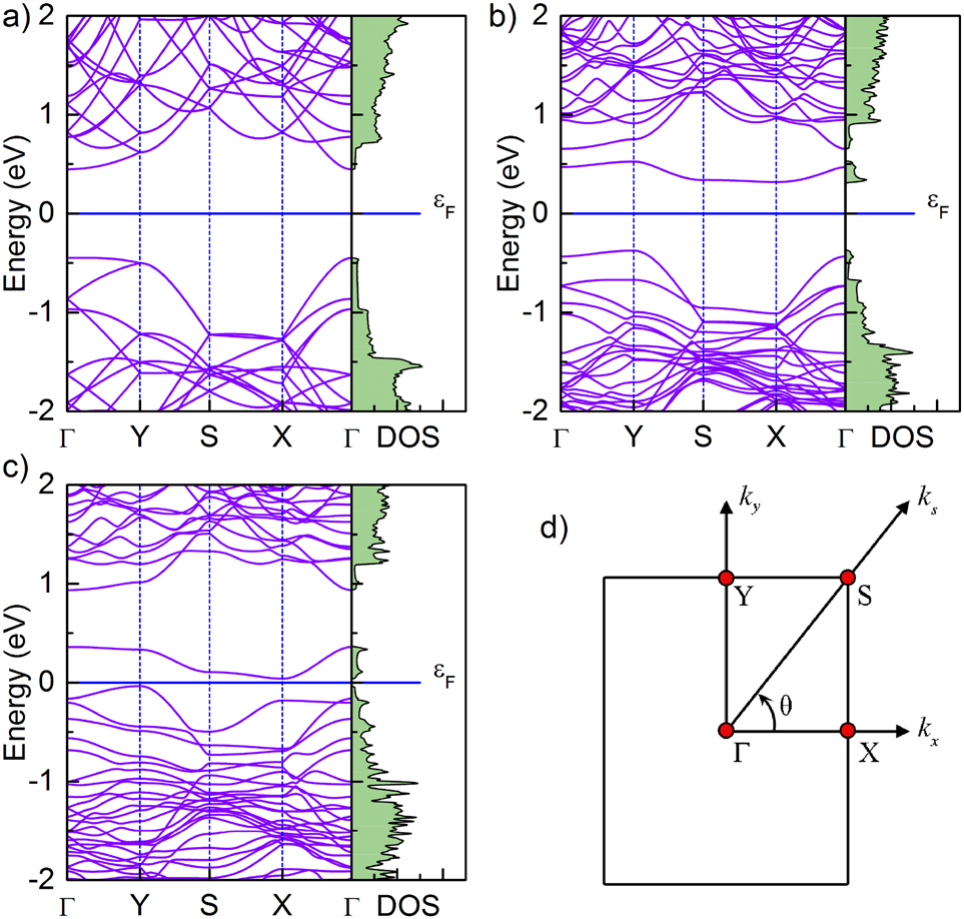
Electronic band structure along the $\Gamma$–Y–S–X–$\Gamma$ direction and density of states (DOS) of (**a**) pristine, (**b**) B-doped, and (**c**) Cu-doped phosphorenes. The Fermi level set to zero is marked with the horizontal blue line. (**d**) The irreducible Brillouin zone of phosphorene with high-symmetry points marked by red circles.

In the next step, the electronic band structures are computed for the substituted systems from which the widths of the bandgaps are determined. As we can see in Fig. 4a, the pristine monolayer phosphorene is a semiconductor with a direct bandgap of 0.90 eV, which is consistent with the results obtained in other papers^62–65^. After calculating the band structures of phosphorene substitutionally doped with different atoms, we found that the electronic properties of the phosphorene can be flexibly manipulated. As an effect of substitutional doping, the electronic structure of phosphorene changes but all investigated systems show semiconducting behavior. P$_{47}$Cu, P$_{47}$F and P$_{47}$Cl are narrow gap semiconductors with the band gap of 0.07, 0.10 and 0.08 meV, respectively (see Fig. 4b–d) for exemplary results of B- and Cu-substitution). It should be noted here, that the phosphorene seems to be very susceptible to this kind of doping. The replacement of only 1 of 48 atoms (about $2\%$) seems to heavily affect both the electronic and structural properties of BP. After introducing substitution, the bandgap widths for Co, B, and Sc substituted phosphorene were 0.42 eV, 0.72 eV, and 0.81 eV, respectively. In B-substituted material, band calculations showed that the valence band maximum (VBM) and conduction band minimum (CBM) are located at the *Y* and *X* points, respectively. In this case, the Fermi level lies within the indirect bandgap near the VBM, which indicates a p-type semiconductor. The Co and Sc substituted materials show a different behavior, as Fermi level lies close to CBM. Therefore, both are n-type semiconductors and Sc and Co act like donors. It is interesting to note that the Cu substituted BP shows a reduction in band gap width. As can be seen from Fig. 4c this is caused by the emergence of the additional band above the Fermi energy level in comparison to pristine BP. For the F and Cl substitutions, the wide to narrow semiconductor transition is also caused by the emergence of the band above the Fermi level.

**Figure 5:**
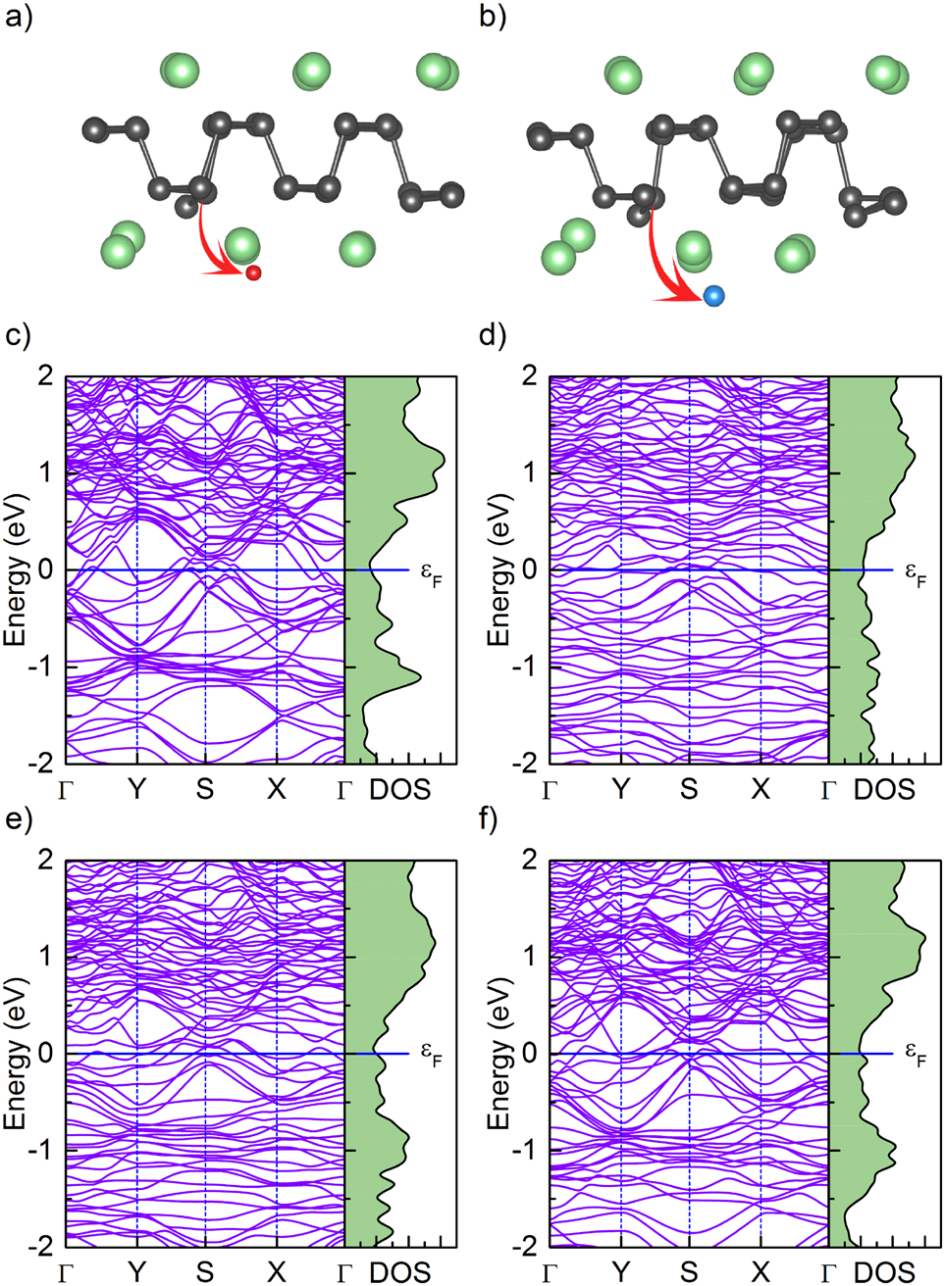
Structural deformation of the (**a**) F- and (**b**) Cl-doped phosphorene lattice at the adsorption of Mg atoms. The gray and green spheres represent P and Mg atoms, respectively. Red arrows indicate the dopants (F and Cl atoms marked with red and blue balls, respectively) pulled out of the plane during the structure optimization. Electronic band structure and density of states of fully Mg covered phosphorene sheet with (**c**) B-, (**d**) Sc-, (**e**) Co-, and f) Cu-substitution in place of one P atom.

To investigate the utility of the above 2D nanomaterials, as anodes for Mg-ion batteries, we analyzed the changes in the geometric and electronic structure of doped monolayer phosphorene upon Mg adsorption above the hollow site. As shown in Fig. 5a and b in the case of F- and Cl-doped BP, we found that the adsorption of 24 Mg adatoms, yields a structural deformation in the phosphorene layer through pulling F or Cl atoms out of the plane during the structure optimization process. Typically, the final results of the relaxation process are sensitive to the starting position of a dopant, therefore, several different starting locations had been carefully checked, nonetheless, relaxation always led to the same result. Such behavior indicates a deep energy minimum related to the resulting dopant position which is out of the phosphorene surface. Therefore, this suggests that F- and Cl-doped phosphorene cannot be considered as an anode material for battery applications because it would prone to the destruction of the entire structure during the ionic diffusion process. In all other cases, the modified phosphorene sheets were stable after the adsorption of Mg atoms on both sides. To investigate the bonding distance of Mg ion adsorbed onto the phosphorene surface we calculated the distance between Mg and three nearest surface atoms, one being always the substituted one (Mg–${\mathrm P^{nearest}}_I$, Mg–${\mathrm P^{nearest}}_{II}$, Mg–Dopant), and the average distance ${\overline{R}}$ between Mg and these nearest atoms from substrate. Such an approach gives better description of surface bonding than giving just the distance between the substituted atom and Mg. This, substitution leads to distortion of neighboring P atoms as dopant radius differs significantly between smallest B (0.84 Å) and largest Sc (1.6 Å) atomic radius. Such deformation is especially visible in the case of Sc, where neighboring P atoms are “swollen” around Sc, which is pushed into the hollow site. The calculated average distances are collected in Table 1. As can be seen in the cases of B, Co, and Cu average distances are smaller in comparison with non-modified pristine material indicating stronger bonding to Mg. This behavior may positively influence the intercalation process of Mg due to the decrease of distance between Mg and BP surface, which could reduce the interlayer volumetric expansion.

The electronic band structures in Fig. 5c–f indicate a metallic behavior of these systems with several bands crossing the Fermi level along many directions and with many available electronic states at the Fermi level.

**Table 1:** Calculated lowest distances (in Å units) between adsorbed Mg and two phosphorene nearest atoms (${\mathrm P^{nearest}}_I$ and ${\mathrm P^{nearest}}_{II}$), lowest distances between adsorbed Mg and dopant (B, Sc, Co, Cu atoms), and the average distance ${\overline{R}}$ between Mg and three nearest substrate atoms.

System	Mg–${\mathrm{P}}^{\mathrm{nearest}}_{\mathrm{I}}$	Mg–Dopant	Mg–${\mathrm{P}}^{\mathrm{nearest}}_{\mathrm{II}}$	${\overline{R}}$
Pristine	2.6860	2.9170	2.9170	2.8400
P$_{47}$B	2.6956	2.7137	2.7519	2.7204
P$_{47}$Co	2.7030	2.7504	2.8520	2.7684
P$_{47}$Cu	2.6913	2.6618	2.7915	2.7148
P$_{47}$Sc	2.6634	3.4964	2.8569	3.0055

The adsorption energy per Mg atom on monolayer phosphorene ($E_a$) is defined as:

2 $$\begin{aligned} E_a=(E_{\mathrm{Mg}_n{\mathrm{P_{47}}+dopant}} - E_{\mathrm{P_{47}}+dopant} - nE_{\mathrm{Mg}})/n, \end{aligned}$$

where $E_{\mathrm{Mg}_n{\mathrm{P_{47}}+dopant}}$, $E_{\mathrm{P_{47}}+dopant}$, and $E_{\mathrm{Mg}}$ are the total energy of the doped $3 \times 4$ phosphorene supercell after adsorption of $n=24$ Mg atoms on both sides, the total energy of free standing doped phosphorene sheet, and the isolated Mg atom, respectively^66^. The negative value of $E_a$ means that the adsorption process is an exothermic reaction and energetically preferable. The resulting adsorption energies are summarized in Table 2. One can see that Sc-doped BP is the most energetically stable adsorption substrate with $E_{a}$ of $-1.357$ eV/Mg. The adsorption energy is directly connected with the diffusion ability of the Mg atoms on the phosphorene surface. The relatively higher absolute value of adsorption energy and lower value of the bond distance between Mg and surface atoms typically means more stability and as a result better diffusion rate of the Mg to the surface^46,67,68^. The calculated $E_{a}$ energies for B, Sc, Co, and Cu substituted material are lower than in the pristine case. This suggests, that the performance of modified electrodes should be better in comparison with pristine material due to the improved stability.

**Figure 6:**
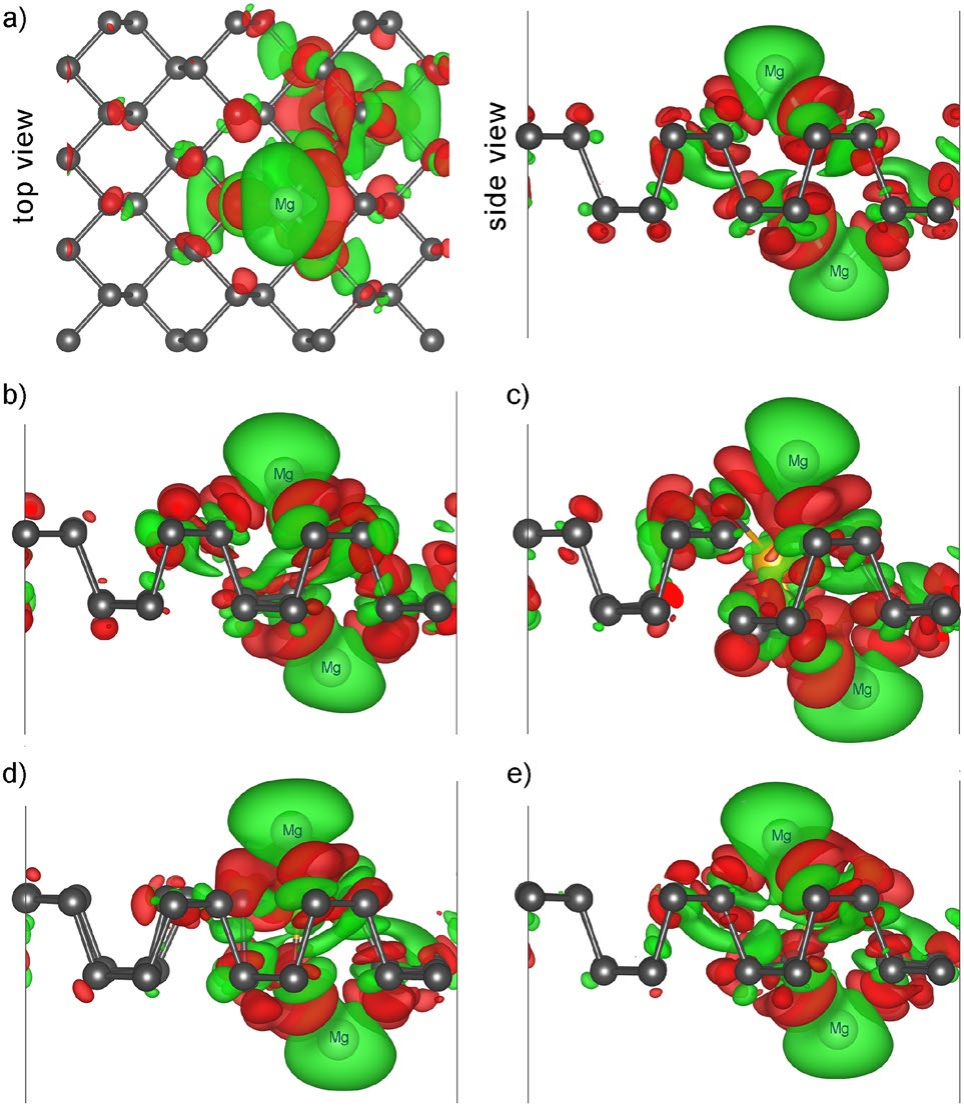
The charge difference plots of two Mg atoms adsorbed on (**a**) pristine BP, (**b**) B-, (**c**) Sc-, (**d**) Co-, and (**e**) Cu-doped phosphorene The loss of electrons is indicated in green and gain of electrons is indicated in red. The isosurface level was set at 0.0007$a_0^{-3}$, where $a{_0}$ is the Bohr radius.

To better understand the bonding property between Mg atoms and doped phosphorene we calculated the charge density difference using the relation:

3 $$\begin{aligned} \Delta \rho = \rho _{\mathrm{substrate+2Mg}}-\rho _{\mathrm{2Mg}}-\rho _{\mathrm{substrate}}, \end{aligned}$$

where $\rho _{\mathrm{substrate+2Mg}}$ and $\rho _{\mathrm{substrate}}$ are the charge densities of substitutionally doped BP system with and without two adsorbed Mg atoms, $\rho _{\mathrm{2Mg}}$ is the charge densities of isolated two Mg atoms. The charge difference plots of B-, Sc-, Co, and Cu-doped phosphorene are shown in Fig. 6, with green and red areas representing the loss and gain of the electrons, respectively.

Charge-depleted regions surround Mg atoms and charge accumulation takes place near the doped phosphorene monolayer, indicating a significant charge transfer from the metal atoms to the substrate^69^. This result manifests that the Mg adsorption forms strong ionic bonding, which means that doped phosphorene may be better anode material for Mg-ion batteries, as such a bonding typically indicates better system stability. From the top view of the pristine phosphorene (Fig. 6a), it is visible, that both Mg atoms are bonded to three neighboring P atoms. The Mg ions after relaxation tend to be closer to one of the nearest P atoms, shifting from the center of the hollow site (see Fig. 5a). However, as can be seen on the charge difference plots this asymmetry only slightly reduces the interaction between P atom located on the opposite side. Therefore, observed charge transfer is delocalized over a larger area, thus improving adsorption stability.

**Figure 7:**
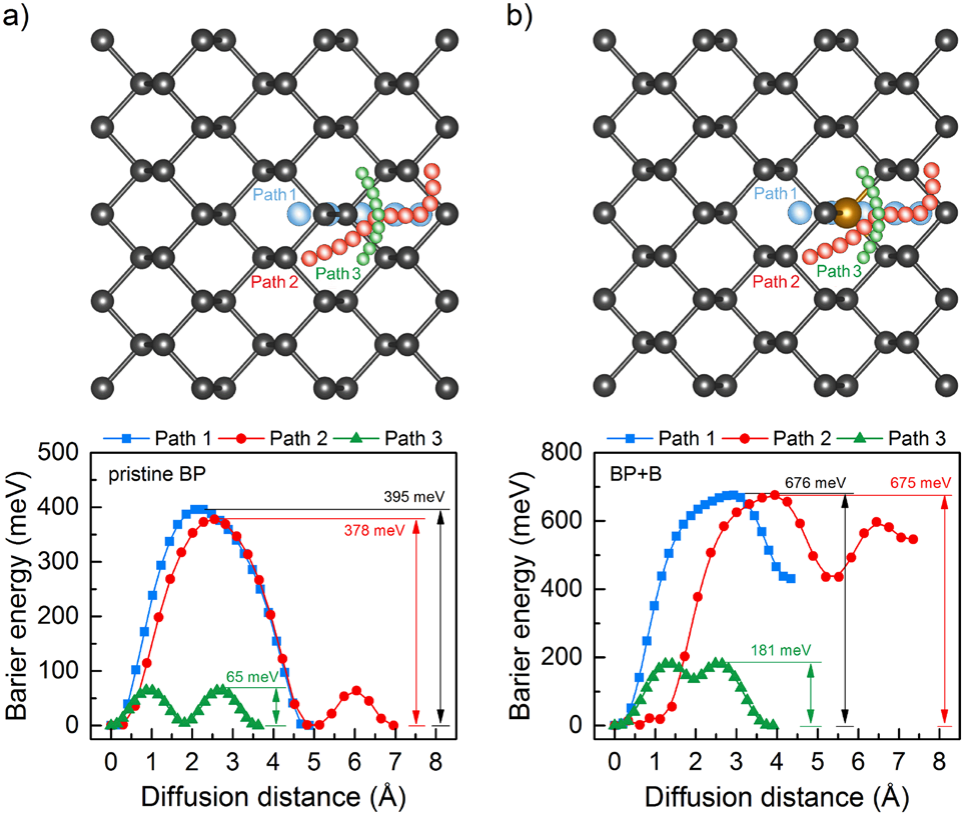
Three possible Mg ion migration paths over pristine (**a**) and B-doped (**b**) phosphorene.

The performance of the electrode material is closely related to the mobility of the adsorbed ions. In general, a lower diffusion barrier means a higher diffusion rate or mobility of ions on the surface of the material. Thus, it is necessary to study the diffusion behavior of Mg ions when the pristine and B-doped BP monolayers are used as the substrate. The migrations of Mg atoms are studied using the CI-NEB method^70^. The results obtained for pristine and B-doped phosphorene are presented in Fig. 7a and b, respectively. The blue, red, and green points represent the selected possible diffusion pathways of the Mg atom. Note, that Path 1 was chosen to be realized on the opposite side of phosphorene relative to Path 2 and Path 3. We found that the optimal Mg migration path is Path 3 with low diffusion barriers (65 meV for pristine BP and 181 meV for B-doped BP), which ensures fast ion diffusion on pristine phosphorene monolayer. In the case o B doped material, the greater energy barrier observed in all studied paths is probably caused by increased bonding strength between Mg and B which may be concluded by analyzing dopant-Mg distance in Table 1 and by greater adsorption energy in respect to P-Mg in pristine material (see $E_{a}$ in Table 2). Thus, mobility over the doped region is decreased. Even though this can be a major disadvantage of B-doped BP monolayer when used as anodes, it does not eliminate this material as long as the remaining parameters are promising.

Let us now turn to the study of electronic properties of substituted material by charge analysis with a single Mg adsorbed onto both sides of phosphorene using the Bader charge package^71,72^. The results of these calculations are presented in Table 2. As can be seen, when only two Mg atoms are adsorbed on both sides of pristine BP, the charge transfer from Mg to the surface is close to 0.92 $e^{-}$. Introducing B rises the observed charge transfer up to 1.1 $e^{-}$ for Mg adsorbed near the substitution. As can be seen, in the case of a single Mg absorbed on both BP sides, each addition resulted in an increased total charge transfer from Mg to the surface ($|\Delta Q_{Mg\uparrow +Mg\downarrow }|$) in respect to pristine phosphorene. The highest charge transfer was obtained for B substituted BP and the lowest value was observed for Co, which still is higher than the one for the pristine phosphorene. Interestingly, the charge transfer from Mg was not always greater on the side where the substitution was close to Mg ($|\Delta Q_{Mg\downarrow }|$). The inverse behavior was observed for Co and Cu substituted materials, so in the cases with the least structural deformations around the substitution.

**Table 2:** Calculated adsorption energy ($E_a$), absolute charge transfer from double-side adsorbed single Mg (Mg$\uparrow$: Mg adsorbed to upper side, Mg$\downarrow$: Mg adsorbed to bottom side), total absolute charge transfer from Mg on both sides $\Delta Q_{Mg\uparrow +Mg\downarrow }$ to substrate, average charge transfer of adsorbed 24 Mg atoms on both sides ($\overline{\Delta Q_{Mg}}$), and storage capacity of BP substitutionally doped by B, Sc, Co and Cu atoms with double-side Mg fully occupancy.

System	$E_{a}$ (eV/Mg)	$|\Delta Q_{Mg\uparrow }|$ ($e^{-}$)	$|\Delta Q_{Mg\downarrow }|$ ($e^{-}$)	$|\Delta Q_{Mg\uparrow +Mg\downarrow }|$ ($e^{-}$)	$\overline{\Delta Q_{Mg}}$ ($e^{-}$)	*C* (mAh/g)
Pristine	− 0.998	0.923	0.924	1.847	0.766	238.0
P$_{47}$B	− 1.229	0.918	1.137	2.055	0.748	234.7
P$_{47}$Sc	− 1.357	0.984	0.996	1.980	0.757	233.6
P$_{47}$Co	− 1.312	0.975	0.907	1.882	0.760	232.9
P$_{47}$Cu	− 1.226	0.993	0.982	1.975	0.752	230.0

In Table 2 an analysis of the average charge transfer ($\overline{\Delta Q_{Mg}}$) between the phosphorene and 24 Mg atoms is shown. Especially in the cases of BP monolayer modified by B, Sc, Co, and Cu atoms giving $\overline{\Delta Q_{Mg}}$ values of 0.748, 0.757, 0.760, and 0.752 $e^{-}$, respectively, which suggests strong adsorption energy. However, when comparing charge transfer with 24 Mg adsorbed atoms to the case with only 2 adsorbed Mg, a drop in the charge transfer from average 1 $e^{-}$/atom to average 0.75 $e^{-}$/atom is observed. This decrease is also visible in the pristine BP case, therefore, it must be related to the number of Mg ions attached to the surface, which most probably is connected with Mg–Mg ionic repulsion becoming significant when the surface is tightly packed with Mg. However, decreasing in average charge transfer is more visible when the surface is doped. In those cases the local charge transfer is increased by 0.2$e^{-}$ but the overall average charge transfer is lower in comparison to pristine BP. The above analysis suggests that to further improve the properties of the material, it is necessary to use more substitutions, which could cause both, decrease in bonding distance between Mg and surface and an increase in charge transfer from Mg to the BP surface. Yet, further calculations are required to confirm this assumption because the interactions occurring in the material are very complex.

The gravimetric capacity, one of the most important parameters characterizing modern energy storage devices, can be calculated using the following equation^73,74^:

4 $$\begin{aligned} C=\frac{\overline{\Delta Q_{Mg}} nF}{M_{\mathrm{P_{47}}+dopant}+nM_{\mathrm{Mg}}} \end{aligned}$$

where $\Delta Q_{Mg}$ is the average charge transfer from each Mg to the phosphorene surface, $M_{\mathrm{P_{47}}+dopant}$ and $M_{\mathrm{Mg}}$ are the molar weights of doped BP supercell and one Mg atom, *n* is the number of Mg atoms adsorbed on the substrate, and *F* is the Faraday constant (26801 mAh/mol).

Our calculation results, collected in Table 2, demonstrate that when the surface of phosphorene is covered by Mg, the capacity reaches its maximum value of 238.0 mAh/g for pristine phosphorene anode. It should be noted here that this value is strongly underestimated due to the specific choice of our system construction. In the case where one Mg ion would be intercalated between two sheets of phosphorene, each ion will give its charge to both surrounding layers, thus increasing overall capacity. As can be seen in each case of doped phosphorene the total capacity decreases, which is a combined effect of reduced average charge transfer and increased mass of dopants (excluding boron). This effect directly indicates that the use of a small percentage of dopant has a negative effect on the electrode capacity. Additionally, our results are found to be smaller than the one of the commercially used graphite anode (372 mAh/g) in Li-ion batteries^3^ but the direct comparison is difficult. More importantly, the local change of electronic properties, caused by substitution, changes the binding energy between Mg and modified surface, which should also alter other important properties of the electrode like the mentioned diffusion rate and material stability.

## Conclusions

The structural stability and electronic properties of B-, F-, Cl-, Sc-, Co-, and Cu-doped phosphorene upon Mg adsorption were studied using DFT calculations. As we showed, the introduction of dopants can affect the local properties of the material, changing many of its parameters. We find that the changing of one P atom with B in black phosphorene increased charge transfer near the substitution region, which is the desired effect. However, on the other side, it decreased charge transfer from Mg adsorbed further from dopant. It suggests that to increase the total charge transfer much more dopant is necessary, perhaps even completely new material with a much higher B/P ratio, combining low volumetric expansion of boron and relatively high capacity of phosphorene, would be desirable. It should be noted, that the reduction in volumetric expansion possible by increasing dopant content can lead to a better life cycle of the battery. Moreover, the improvement of anode properties can be reached not only by increasing its theoretical capacity but due to increased binding energy between Mg and substituted BP. It can be achieved through the modification of the material, however, local structure deformation should be taken into account. It is not clear yet, whether such a distortion would provide a better performance, but because such modifications (excluding Cl and F substitutions) make the material more stable, it seems promising. Therefore, ab initio molecular dynamics^75^ calculations could be helpful for further evaluation of anode working stability.

## References

[1] Wright DR, Garcia-Araez N, Owen JR (2018). Review on high temperature secondary Li-ion batteries. Energy Proc..

[2] Nishi Y (2001). Lithium ion secondary batteries; past 10 years and the future. J. Power Sour..

[3] Northcott CJ, Stein MB (2001). Issues and challenges facing rechargeable lithium batteries. Nature.

[4] Mauger A, Julien CM, Paolella A, Armand M, Zaghib K (2018). A comprehensive review of lithium salts and beyond for rechargeable batteries: Progress and perspectives. Mater. Sci. Eng. R Rep..

[5] Jiang HR (2016). A promising anode material offering high specific capacity and high rate capability for lithium-ion batteries. Nano Energy.

[6] Kasavajjula U, Wang C, John Appleby A (2007). Nano- and bulk-silicon-based insertion anodes for lithium-ion secondary cells. J. Power Sour..

[7] Gil-Alana LA, Monge M (2019). Lithium: Production and estimated consumption. Evidence of persistence. Resour. Policy.

[8] Sterba J, Krzemień A, Pedro Riesgo F, Carmen Escanciano G-M, Gregorio Fidalgo V (2019). Lithium mining: Accelerating the transition to sustainable energy. Resour. Policy.

[9] Sun X, Hao H, Hartmann P, Liu Z, Zhao F (2019). Supply risks of lithium-ion battery materials: An entire supply chain estimation. Mater. Today Energy.

[10] Liu D, Gao X, An H, Qi Y, Sun X, Wang Z, Chen Z, An F, Jia N (2019). Supply and demand response trends of lithium resources driven by the demand of emerging renewable energy technologies in China. Resour. Conserv. Recycl..

[11] Hong X (2019). Nonlithium metal-sulfur batteries: Steps toward a leap. Adv. Mater..

[12] Larcher D, Tarascon JM (2015). Towards greener and more sustainable batteries for electrical energy storage. Nat. Chem..

[13] Rasul S, Suzuki S, Yamaguchi S, Miyayama M (2012). Synthesis and electrochemical behavior of hollandite MnO2/acetylene black composite cathode for secondary Mg-ion batteries. Solid State Ion..

[14] Singh N, Arthur TS, Ling C, Masaki M, Mizuno F (2013). A high energy-density tin anode for rechargeable magnesium-ion batteries. Chem. Commun..

[15] Vogt P (2012). Silicene: Compelling experimental evidence for graphenelike two-dimensional silicon. Phys. Rev. Lett..

[16] Nouri N, Rashedi G (2018). Band structure of monolayer of graphene, silicene and silicon-carbide including a lattice of empty or filled holes. J. Semicond..

[17] Yankowitz M, Ma Q, Jarillo-Herrero P, LeRoy BJ (2019). van der Waals heterostructures combining graphene and hexagonal boron nitride. Nat. Rev. Phys..

[18] Al Hassan MR, Sen A, Zaman T, Mostari MS (2019). Emergence of graphene as a promising anode material for rechargeable batteries: A review. Mater. Today Chem..

[19] Zhao R, Qian Z, Liu Z, Zhao D, Hui X, Jiang G, Wang C, Yin L (2019). Molecular-level heterostructures assembled from layered black phosphorene and ti3c2 mxene as superior anodes for high-performance sodium ion batteries. Nano Energy.

[20] Ying W, Yan Yu (2019). 2d material as anode for sodium ion batteries: Recent progress and perspectives. Energy Storage Mater..

[21] Kasprzak GT, Gruszka KM, Durajski AP (2021). Theoretical investigation of c3n monolayer as anode material for li/na-ion batteries. Acta Phys. Pol. A.

[22] Yi Y, Yu XF, Zhou W, Wang J, Chu PK (2017). Two-dimensional black phosphorus: Synthesis, modification, properties, and applications. Mater. Sci. Eng. R Rep..

[23] Qiao J (2014). High-mobility transport anisotropy and linear dichroism in few-layer black phosphorus. Nat. Commun..

[24] Mukherjee S, Singh G (2019). Two-dimensional anode materials for non-lithium metal-ion batteries. ACS Appl. Energy Mater..

[25] Liu W, Zhi H, Xuebin Yu (2019). Recent progress in phosphorus based anode materials for lithium/sodium ion batteries. Energy Storage Mater..

[26] Liu C, Xinpeng Han Yu, Cao SZ, Zhang Y, Sun J (2019). Topological construction of phosphorus and carbon composite and its application in energy storage. Energy Storage Mater..

[27] Yabuuchi N (2014). Phosphorus electrodes in sodium cells: Small volume expansion by sodiation and the surface-stabilization mechanism in aprotic solvent. ChemElectroChem.

[28] Youngjin K (2013). An amorphous red phosphorus/carbon composite as a promising anode material for sodium ion batteries. Adv. Mater..

[29] Kulish VV, Malyi OI, Persson C, Ping W (2015). Phosphorene as an anode material for na-ion batteries: A first-principles study. Phys. Chem. Chem. Phys..

[30] Kulish VV, Malyi OI, Persson C, Ping W (2015). Adsorption of metal adatoms on single-layer phosphorene. Phys. Chem. Chem. Phys..

[31] Liu X, Wen Y, Chen Z, Shan B, Chen R (2015). A first-principles study of sodium adsorption and diffusion on phosphorene. Phys. Chem. Chem. Phys..

[32] Zhao S, Kang W, Xue J (2014). The potential application of phosphorene as an anode material in Li-ion batteries. J. Mater. Chem. A.

[33] Han X, Liu C, Sun J, Sendek AD, Yang W (2018). Density functional theory calculations for evaluation of phosphorene as a potential anode material for magnesium batteries. RSC Adv..

[34] Liu C, Wang Y, Sun J, Chen A (2020). A review on applications of layered phosphorus in energy storage. Trans. Tianjin Univ..

[35] Gazzari S, Cortés-Arriagada D (2020). Uptake of formaldehyde onto doped phosphorene nanosheets: A cluster dft study of single and co-adsorption states. J. Alloys Compd..

[36] Zhu J, Zhang J, Shengrui X, Hao Y (2017). Unintentional doping effects in black phosphorus by native vacancies in h-BN supporting layer. Appl. Surf. Sci..

[37] Jun D, Xiao Cheng Z (2014). Structure and stability of two dimensional phosphorene with [double bond, length as m-dash]O or [double bond, length as m-dash]NH functionalization. RSC Adv..

[38] Sun X, Wang Z (2018). Sodium adsorption and diffusion on monolayer black phosphorus with intrinsic defects. Appl. Surf. Sci..

[39] Boukhvalov DW, Rudenko AN, Prishchenko DA, Mazurenko VG, Katsnelson MI (2015). Chemical modifications and stability of phosphorene with impurities: A first principles study. Phys. Chem. Chem. Phys..

[40] Sun J, Lee H-W, Pasta M, Yuan H, Zheng G, Sun Y, Li Y, Cui Y (2015). A phosphorene-graphene hybrid material as a high-capacity anode for sodium-ion batteries. Nat. Nanotechnol..

[41] Liu H (2017). Bridging covalently functionalized black phosphorus on graphene for high-performance sodium-ion battery. ACS Appl. Mater. Interfaces.

[42] Butt MH (2021). Cu-doped phosphorene as highly efficient single atom catalyst for co oxidation: A dft study. Mol. Catal..

[43] Marjani A, Ghashghaee M, Ghambarian M, Ghadiri M (2021). Scandium doping of black phosphorene for enhanced sensitivity to hydrogen sulfide: Periodic dft calculations. J. Phys. Chem. Solids.

[44] Cortés-Arriagada D, Ortega DE (2020). Removal of arsenic from water using iron-doped phosphorene nanoadsorbents: A theoretical dft study with solvent effects. J. Mol. Liq..

[45] Hanif A (2021). Tuning the optoelectronic properties of superalkali doped phosphorene. J. Mol. Graph. Modell..

[46] Luo Yi (2018). Adsorption of transition metals on black phosphorene: a first-principles study. Nanoscale Res. Lett..

[47] Henry L, Svitlyk V, Mezouar M, Sifré D, Garbarino G, Ceppatelli M, Serrano-Ruiz M, Peruzzini M, Datchi F (2020). Anisotropic thermal expansion of black phosphorus from nanoscale dynamics of phosphorene layers. Nanoscale.

[48] Parr RG, Yang W (1989). Density-Functional Theory of Atoms and Molecules.

[49] Giannozzi P (2009). Quantum espresso: A modular and open-source software project for quantum simulations of materials. J. Phys. Condens. Matter.

[50] Giannozzi P (2017). Advanced capabilities for materials modelling with Quantum ESPRESSO. J. Phys. Condens. Matter.

[51] Billeter SR, Curioni A, Andreoni W (2003). Efficient linear scaling geometry optimization and transition-state search for direct wavefunction optimization schemes in density functional theory using a plane-wave basis. Comput. Mater. Sci..

[52] Zhu Z, Tománek D (2014). Semiconducting layered blue phosphorus: A computational study. Phys. Rev. Lett..

[53] Li Y, Yang S, Li J (2014). Modulation of the electronic properties of ultrathin black phosphorus by strain and electrical field. J. Phys. Chem. C.

[54] Sibari A, Marjaoui A, Lakhal M, Kerrami Z, Kara A, Benaissa M, Ennaoui A, Hamedoun M, Benyoussef A, Mounkachi O (2018). Phosphorene as a promising anode material for (li/na/mg)-ion batteries: A first-principle study. Sol. Energy Mater. Sol..

[55] Srivastava P (2015). Tuning the electronic and magnetic properties of phosphorene by vacancies and adatoms. J. Phys. Chem. C.

[56] Durajski AP, Auguscik AE, Szczesniak R (2020). Tunable electronic and magnetic properties of substitutionally doped graphene. Phys. E.

[57] Wang G, Pandey R, Karna SP (2015). Effects of extrinsic point defects in phosphorene: B, C, N, O, and F adatoms. Appl. Phys. Lett..

[58] Hashmi A, Hong J (2015). Transition metal doped phosphorene: First-principles study. J. Phys. Chem. C.

[59] Gazzari S, Wrighton-Araneda K, Cortés-Arriagada D (2020). A first-principles description of the stability of transition-metal doped phosphorene nanosheets. Surf. Interfaces.

[60] Kim GH, Jeong S (2015). Change of electronic structures by dopant-induced local strain. Sci. Rep..

[61] Khalatbari H, Izadi Vishkayi S, Oskouian M, Rahimpour Soleimani H (2021). Band structure engineering of NiS2 monolayer by transition metal doping. Sci. Rep..

[62] Wang C, Xia Q, Nie Y, Guo G (2015). Strain-induced gap transition and anisotropic dirac-like cones in monolayer and bilayer phosphorene. J. Appl. Phys..

[63] Sa B, Li Y-L, Qi J, Ahuja R, Sun Z (2014). Strain engineering for phosphorene: The potential application as a photocatalyst. J. Phys. Chem. C.

[64] Liu H, Neal AT, Zhu Z, Luo Z, Xianfan X, Tománek D, Ye PD (2014). Phosphorene: An unexplored 2d semiconductor with a high hole mobility. ACS Nano.

[65] Fei R, Yang L (2014). Strain-engineering the anisotropic electrical conductance of few-layer black phosphorus. Nano Lett..

[66] Zhang C (2017). The prospects of phosphorene as an anode material for high-performance lithium-ion batteries: A fundamental study. Nanotechnology.

[67] Boukhvalov DW, Rudenko AN, Prishchenko DA, Mazurenko VG, Katsnelson MI (2015). Chemical modifications and stability of phosphorene with impurities: A first principles study. Phys. Chem. Chem. Phys..

[68] Durajski AP, Gruszka KM, Niegodajew P (2020). First-principles study of a substitutionally doped phosphorene as anode material for na-ion batteries. Appl. Surf. Sci..

[69] Mukherjee S, Kavalsky L, Singh CV (2018). Ultrahigh storage and fast diffusion of na and k in blue phosphorene anodes. ACS Appl. Mater. Interfaces.

[70] Henkelman G, Uberuaga BP, Jónsson H (2000). A climbing image nudged elastic band method for finding saddle points and minimum energy paths. J. Chem. Phys..

[71] Henkelman G, Arnaldsson A, Jonsson H (2006). A fast and robust algorithm for bader decomposition of charge density. Comput. Mater. Sci..

[72] Tang W, Sanville E, Henkelman G (2009). A grid-based bader analysis algorithm without lattice bias. J. Phys. Condens. Matter.

[73] Liu H, Dong H, Yujin Ji L, Wang TH, Li Y (2019). The adsorption, diffusion and capacity of lithium on novel boron-doped graphene nanoribbon: A density functional theory study. Appl. Surf. Sci..

[74] Zhou L, Hou ZF, Gao B, Frauenheim T (2016). Doped graphenes as anodes with large capacity for lithium-ion batteries. J. Mater. Chem. A.

[75] Tuckerman ME (2002). Ab initio molecular dynamics: Basic concepts, current trends and novel applications. J. Phys. Condens. Matter.

